# Spontaneous Mutation Reveals Influence of Exopolysaccharide on *Lactobacillus johnsonii* Surface Characteristics

**DOI:** 10.1371/journal.pone.0059957

**Published:** 2013-03-27

**Authors:** Nikki Horn, Udo Wegmann, Enes Dertli, Francis Mulholland, Samuel R. A. Collins, Keith W. Waldron, Roy J. Bongaerts, Melinda J. Mayer, Arjan Narbad

**Affiliations:** 1 Department of Gut Health and Food Safety, Institute of Food Research, Norwich, Norfolk, United Kingdom; 2 Department of Food Engineering, Bayburt University, Bayburt, Turkey; 3 Proteomics Unit, Institute of Food Research, Norwich, Norfolk, United Kingdom; 4 Department of Food and Health, Institute of Food Research, Norwich, Norfolk, United Kingdom; 5 Analytical Sciences Unit, Institute of Food Research, Norwich, Norfolk, United Kingdom; Institut Pasteur de Lille, France

## Abstract

As a competitive exclusion agent, *Lactobacillus johnsonii* FI9785 has been shown to prevent the colonization of selected pathogenic bacteria from the chicken gastrointestinal tract. During growth of the bacterium a rare but consistent emergence of an altered phenotype was noted, generating smooth colonies in contrast to the wild type rough form. A smooth colony variant was isolated and two-dimensional gel analysis of both strains revealed a protein spot with different migration properties in the two phenotypes. The spot in both gels was identified as a putative tyrosine kinase (EpsC), associated with a predicted exopolysaccharide gene cluster. Sequencing of the *epsC* gene from the smooth mutant revealed a single substitution (G to A) in the coding strand, resulting in the amino acid change D88N in the corresponding gene product. A native plasmid of *L. johnsonii* was engineered to produce a novel vector for constitutive expression and this was used to demonstrate that expression of the wild type *epsC* gene in the smooth mutant produced a reversion to the rough colony phenotype. Both the mutant and *epsC* complemented strains had increased levels of exopolysaccharides compared to the wild type strain, indicating that the rough phenotype is not solely associated with the quantity of exopolysaccharide. Another gene in the cluster, *epsE*, that encoded a putative undecaprenyl-phosphate galactosephosphotransferase, was deleted in order to investigate its role in exopolysaccharide biosynthesis. The Δ*epsE* strain exhibited a large increase in cell aggregation and a reduction in exopolysaccharide content, while plasmid complementation of *epsE* restored the wild type phenotype. Flow cytometry showed that the wild type and derivative strains exhibited clear differences in their adhesive ability to HT29 monolayers in tissue culture, demonstrating an impact of EPS on surface properties and bacteria-host interactions.

## Introduction

*Lactobacillus johnsonii* FI9785 is a poultry-derived Gram-positive bacterium which has been fully sequenced [1]. It has been shown to act as a probiotic, being effective in excluding the pathogen *Clostridium perfringens* from specific pathogen-free chicks during an *in vivo* challenge study [2]. *C. perfringens* causes necrotic enteritis in poultry and food poisoning in humans and therefore has an important impact on both animal health and the safety of the food chain [3]. The mechanism of exclusion is not yet defined. Protection requires pre-inoculation with *L. johnsonii*, so it could rely on competition for adhesion sites or selected nutrients, or there could be more specific interactions between the gut bacteria [4]. Competition studies have suggested that reduction of pathogen adhesion by lactobacilli is mediated by steric hindrance [5] and *L. johnsonii* NCC533 (La1) has been shown to be able to bind to the same carbohydrate moieties as important enteric pathogens, reinforcing the theory that competition for adhesion sites may contribute to probiotic activity [6].

Bacteria need to survive a range of adverse conditions both in the environment and during transit in the gastrointestinal tract. The presence of stresses such as acid and bile, oxidative and osmotic stress, competing microbes and the host immune response combine with factors such as flow rate to present significant challenges to effective colonisation [4]. The surface characteristics of lactobacilli are expected to contribute in several ways to their interactions with the host gastrointestinal tract and the gut microbiota, affecting their survival, adherence to the host tissue and interactions with themselves and with other bacteria [4], [7]. Exopolysaccharides (EPS) can have important influences on these processes and on the colonisation of the host [8], [9], [10], [11]. Lactic acid bacteria produce both homopolysaccharides and heteropolysaccharides, which can remain bound to the bacterium or be released into the surrounding environment [12], [13]. The use of mutants has shown that EPS production can affect aggregation, biofilm formation, adhesion and survival [10], [11], [14]. Endogenous Lactobacilli have been demonstrated to exist in biofilm-like communities in the avian and murine guts, while exogenously applied *Lactobacillus* spp. had difficulty establishing themselves [15]. This work suggests that factors that enhance biofilm formation and adhesion will be important both for the establishment of the native flora and for potential probiotics, to improve their ability to compete and if possible colonise. Consequently, bacterial surface properties and EPS quantities and/or qualities are expected to play an important role in colonisation and survival in the host.

Heteropolymeric EPS synthesis is normally encoded by a gene cluster of commonly 8–17 genes, encompassing conserved genes for regulation, chain length determination, repeat unit biosynthesis, polymerisation and export [8], [13], [14], [16], [17], [18]. The genome of *L. johnsonii* FI9785 includes a putative 14 gene *eps* cluster whose products show homology to a number of enzymes involved in EPS production [1]. Deletion of a similar cluster in *L. johnsonii* NCC533 removed the ability to produce EPS [19] and resulted in a slight increase in persistence in the murine gut [9].

An aberrant smooth colony morphology was noted in *L. johnsonii* FI9785 plate cultures. The primary objective of this study was to investigate the cause of this phenotype and to assess its effect on the morphology and adhesive behaviour of the cells.

## Materials and Methods

### Bacterial strains, growth conditions and microscopy

*L. johnsonii* FI9785 and *Lactobacillus gasseri* NCIMB 11718 (NCIMB Ltd., Aberdeen) were grown in De Man Rogosa Sharpe medium (MRS, Oxoid) using 2% glucose as a carbon source, at 37°C. *Escherichia coli* MC1022 was grown in L broth at 37°C with shaking. Plasmids were selected and maintained using chloramphenicol at 7.5 µg/ml or erythromycin at 400 µg/ml and 10 µg/ml for *E. coli* and *L. johnsonii* respectively. Bacterial strains and plasmids produced and used in this study are listed in Table 1. Preparation of cells for microscopy and scanning electron microscopy (SEM) was performed as described previously [20]. The presence or absence of pili was determined using transmission electron microscopy (TEM) and negative staining with uranyl acetate. Bacterial suspensions were placed onto pre-coated TEM grids (Agar Scientific, UK), stained with 2% uranyl acetate and fixed for 2 h in vapour from 25% glutaraldehyde. Samples were examined with a FEI Tecnai G2 20 Twin transmission electron microscope at 200 kV.

**Table 1:** Mutant and complemented strains.

Strain	Description	Plasmid
FI9785	Wild type, rough	-
FI10386	Smooth mutant	-
FI10774	Smooth mutant containing empty vector control	pFI2560
FI10773	Smooth mutant co-expressing wild type *epsC*	pFI2660, *espC*
FI10772	Smooth mutant co-expressing mutant *epsC^D88N^*	pFI2659, *epsC^D88N^*
FI10844	*epsE* knockout mutant	-
FI10878	*epsE* knockout mutant complemented with *epsE*	pFI2721, *epsE*
FI10879	*epsE* knockout mutant containing *epsE* antisense control	pFI2722, *epsEAS*

### 2-D gel proteomic analysis

Total soluble protein was isolated from cultures of *L. johnsonii* FI9785 and the smooth mutant FI10386. Cultures were grown in 130 ml MRS to mid-exponential phase (OD_600_ of c. 2) and sample volumes equivalent to 30 OD_600_ units were harvested by centrifugation for 10 min at 3000× g and 4°C. Cells were washed twice with 40 mM Tris HCl (pH 7.4) before being bead beaten in 180 µl protein extraction buffer (40 mM Tris HCl pH 7.4, 3 mM MgCl_2_, 2.5 µl/ml protease inhibitor cocktail (Sigma), 5 U/ml DNase I, 5 µg/ml RNase A) with 0.1 mm glass beads (Sigma) using a FastPrep FP120 cell disrupter (Savant) with 4×30 s bursts on speed setting 6 and incubating on ice for 10 min between bursts. A further 150 µl buffer was added and cell debris/membranes were removed by centrifugation at 13,000× g for 30 min at 4°C. The resulting supernatant constituting the soluble protein extract was flash frozen in liquid nitrogen in aliquots and stored at −70°C.

Proteomic analysis by 2-D gel electrophoresis and MALDI-TOF was performed as described previously [21]. Briefly, 100 µg proteins were isoelectric focussed on 18 cm pH 4–7 IPG strips (APBiotech) for 85 kVh and electrophoresed in the second dimension using 10% Duracryl gels in an Investigator system (Millipore) followed by staining with Sypro Ruby. The stained gels were visualised at 100 µm using a Pharos FX+ laser scanning fluorescent imager (BioRad) and Quantity One software (BioRad). Gels were compared using Proteomweaver software (Definiens version 3.0.1.0). Selected spots were picked and trypsin-digested using the ProPick Spot Picker (Genomic Solutions) and ProGest protein digestion robot (Genomic Solutions) prior to peptide mass fingerprinting on an Ultraflex II MALDI-TOF-TOF (Bruker) using an offline version of Mascot (Matrix Sciences) searching against *Lactobacillus johnsonii* sequences in the Uniprot (sptrembl20121031) database at 50 ppm. Theoretical isoelectric point (pI) and molecular weight (MW) of proteins were calculated using http://web.expasy.org/compute_pi/ [22]. The theoretical mass of tryptic peptides was calculated using http://web.expasy.org/peptide_mass/ [23].

### Bioinformatic and molecular analysis of *eps* genes

Putative gene functions of open reading frames (ORFs) in the *eps* cluster identified previously [1] were assigned by homology to domains and proteins provided by BlastP searches [24]. Amino acid alignments were performed using ClustalW in Vector NTI (Invitrogen). All molecular biology procedures were performed as recommended by the manufacturer. A 3748 bp fragment covering 4 predicted orfs *epsDCBA* was amplified by PCR from *L. johnsonii* smooth variant FI10386 genomic DNA, using the primer pair EPS_5′ (5′-ACGGTATCCTACACGTCC-3′) and EPS_3′ (5′-AAGAGCCGGATTTTTGCC-3′). The product was sequenced in full using the BigDye Terminator v3.1 Cycle Sequencing Kit (Life Technologies Ltd, UK).

### Construction of *L. johnsonii* expression vector pFI2560

A small cryptic plasmid from *L. johnsonii* FI9785 (p9785S [25]) was modified to provide a flexible expression vector. Cloning was performed using *L. gasseri* NCIMB 11718 as the cloning host; following sequence confirmation the final vector was transformed into *L. johnsonii* FI9785, all as described previously [25]. The primer pair p194*Fsp*I (5′-TGCGCACCCATTAGTTCAACAAACG-3′) and p195*Msc*I (5′-CCAACTAACGGGGCAGGTTAGTGAC-3′) (altered nucleotides to create restriction sites are underlined throughout) was used to amplify an 885 bp fragment carrying the chloramphenicol resistance gene from pUK200 [26]. This was cloned into the *Msc*I site of p9785S, regenerating an *Msc*I site downstream of the gene. Next a 97 bp *Eco*RV/RBS/*Nco*I/*Sca*I/*Eco*47III translation fusion linker fragment constructed by the annealing of the primer pair pTF_linker_5′ (5′-AGCGCTTAGCTATCTAAGTACTCCATGGACTGAACCTCCTTTCTGATATC-3′) and pTF_linker_3′ (5′-GATATCAGAAAGGAGGTTCAGTCCATGGAGTACTTAGATAGCTAAGCGCT-3′) was cloned into this *Msc*I site. The *brnQ* terminator fragment from pUK200 was excised with *Xba*I and *Xho*I and blunt ended with T4 DNA polymerase before being subcloned into the new *Eco*47III site provided by the translation fusion linker. The promoter from the *apf1* gene (aggregation promoter factor protein 1; LGAS_1547 in NC_008530) was PCR amplified from *L. gasseri* NCIMB 11718 genomic DNA using the primer pair P*apf1*_F (5′-AGTTCTTAGCTCCTATTTTTTTGCCC-3′) and P*apf1*_R (5′-TTGATAAATTCGATTTGAATTATTTGTTTCGTC-3′). The 208 bp product was cloned into the *Eco*RV site upstream of the ribosome binding site (RBS), ensuring that any gene cloned in frame with the start codon contained in the *Nco*I site would be constitutively expressed at a high level.

### Mutant construction by gene deletion and complementation

The *epsC* and the mutant *epsC^D88N^* genes were PCR amplified respectively from the *L. johnsonii* wild type (FI9785) and the smooth variant (FI10386), using genomic DNA templates together with the primer pair *epsC_Nco*I_5′ (5′-ATCCATGGGATTGTTTAATAGACG-3′) and *epsC*_3′ (5′-TTATTTATTACTTCGTTTCTGTATC-3′). Subsequently each 784 bp PCR product was digested with *Nco*I before being cloned into *Nco*I and *Sca*I-digested pFI2560 to produce the complementation plasmids pFI2660 and pFI2659, respectively. For the deletion of the *epsE* gene from the chromosome of *L. johnsonii* FI9785 the thermosensitive vector pG^+^host9 [27] was employed. Using overlap extension PCR a knockout cassette was produced consisting of flanking sequences from the *epsD* gene followed by a partial sequence of *FI9785_1178*, thereby deleting the *epsE* gene. The *epsD* sequence was PCR amplified with the primer pair KO_F (5′-CTGTCTTTCCAAGTCAAGAAG-3′) and Splice_R (5′-CTAATATGCACTATTCGCATTTAAAACTCTATCCC-3′) and the *FI9785_1178* sequence was amplified using the primer pair KO_R (5′-CCTACCATTCCAATAACATAAC-3′) and Splice_F (5′-GGGATAGAGTTTTAAATGCGAATAGTGCATATTAG-3′), both reactions using FI9785 genomic DNA as the template. The products from these two reactions were then used as templates for the overlap extension PCR together with the primer pair KO_F and KO_R to produce a 1209 bp product. Following restriction using internal *Hind*III and *Bgl*II sites, the 1123 bp digested product was blunt ended and then cloned into *Sma*I-restricted pG^+^host9 to create pFI2702. This construction was performed using *E. coli* MC1022 as the cloning host. Following sequence confirmation and the transformation of *L. johnsonii* FI9785, the method of gene replacement was performed as described by Maguin *et al* [27] using 30°C as the permissive temperature and 42°C as the non-permissive temperature to generate *L. johnsonii* Δ*epsE*, FI10844. The integrity of the gene deletion was confirmed by sequencing this region of the chromosome in full.

For complementation purposes, the *epsE* gene was PCR amplified from FI9785 genomic DNA using the primer pair epsE_5′ (5′-ATGGCACAAGAGGTTAAAAAGG-3′) and epsE_3′ (5′-CTAATATGCACTATTCGGATG-3′) to produce a 657 bp product stretching from the second codon to the stop codon. This product was cloned in both sense and antisense orientations into pFI2560 that had been restricted with *Nco*I and then end-filled to generate pFI2721 and pFI2722 respectively, re-creating the initial methionine start codon in the sense orientation plasmid (pFI2721) only. Transformation of the *L. johnsonii* Δ*epsE* strain FI10844 with pFI2721 and pFI2722 resulted in FI10878 and FI10879 respectively.

### Isolation of exopolysaccharide

EPS was isolated using an adaptation of published methods [28], [29]. Bacteria were grown in 500 ml cultures, inoculated at 1% (v/v) with an overnight culture then incubated at 37°C for 2 d. Samples were agitated vigorously to disperse aggregates and samples taken for cell enumeration by plating. Cells were harvested by centrifugation at 6000× g for 30 min at 4°C and washed twice with PBS, with the initial supernatant being retained. The washed bacterial pellet was resuspended in 50 ml 0.05 M EDTA to extract the capsular EPS. The mixture was incubated with gentle agitation for 4 h at 4°C then centrifuged at 6000× g for 30 min at 4°C. An equal volume of ethanol was added to the supernatant to precipitate the isolated EPS from bacterial cell pellets. As a small proportion of the capsular EPS remained in the culture supernatant and washes from the original centrifugations, this supernatant was retained and remaining EPS was harvested by the addition of an equal volume of ethanol followed by overnight precipitation at 37°C. After this step the same procedures were followed for EPS isolation from either bacterial pellets or the culture supernatants. Samples were centrifuged at 10000× g for 30 min at 4°C and the pellet of the precipitates was retained. The sample was resuspended in water with gentle heating (50°C) and EPS was recovered by precipitation upon the addition of 2 volumes of chilled ethanol. After centrifugation at 10000× g for 30 min at 4°C the resulting EPS was resuspended in distilled water with gentle heating (less than 50°C) followed by dialysis for 72 h (12000–14000-Da visking dialysis membrane, Medicell International, UK) at 4°C, with two changes of H_2_O per day. The contents of the dialysis tubing were freeze-dried to provide EPS. This was further purified by dissolving in 10% TCA and stirring overnight. The precipitated protein was removed by centrifugation at 10000× g for 15 min at 4°C. The pH of the supernatant was adjusted to 7 with 1 M NaOH and EPS was precipitated again with 2 volumes of chilled ethanol. The pellet was dissolved in distilled water and then lyophilized by freeze drying. The EPS samples were stored at 4°C for further analysis.

### Quantification of EPS production

To compare the EPS production of *L. johnsonii* strains, total sugar analysis was carried out by gas chromatography (GC). The pellet and supernatant EPS samples and media controls were hydrolysed to monosaccharides using a modified Saeman hydrolysis (dispersion in 72% H_2_SO_4_ with periodic agitation for 3 h at room temperature followed by dilution to an acid concentration of 1 M H_2_SO_4_ and then further hydrolysis for a total of 2.5 h at 100°C in a hot block). Hydrolysates were made alkaline, reduced, acidified using acetic acid and then derivatized as their alditol acetates as described previously [30] and analysed by capillary GC using a Perkin-Elmer Autosystem XL with an RTX-225 column (Restek, US) with flame ionization detection. Samples were assayed for their levels of rhamnose, fucose, arabinose, xylose, mannose, galactose and glucose using an internal standard of 2-deoxy glucose for calibration purposes. The total amount of EPS from both the bacterial cell pellet and the culture supernatant was combined and expressed as µg EPS per 10^9^ cells.

### Tissue culture growth and adhesion assays

The HT29 cell line (human colon adenocarcinoma, ATCC HTB-38™, LGC) was maintained in tissue culture medium (Dulbecco's modified eagle medium (DMEM, Sigma) supplemented with 10% heat inactivated foetal calf serum (Invitrogen) and 1% MEM non essential amino acids (Sigma)) with 1% Penicillin/Streptomycin (Sigma) in T75 flasks at 37°C in 5% CO_2_. After 3–4 d when cells were at 80% confluence, cells were released with 0.25% trypsin-EDTA (Sigma) and subcultured as recommended (LGC, UK). For adhesion assays cells were grown in 24-well plates (TPP, USA) at a seeding density of c. 6×10^4^ cells/cm^2^ and cultured for 2 d until confluent.

Adhesion assays were performed with *L. johnsonii* strains utilising flow cytometry (FCM) for the accurate quantification of bacterial numbers. Strains were grown overnight in MRS, with chloramphenicol selection for strains containing expression vectors, then harvested by centrifugation. After two washes with 20 ml PBS, cells were resuspended in PBS to an OD_600_ of 1.0, with vortexing to disperse any aggregates. Cells were then diluted in DMEM to a concentration of 1×10^7^ cells/ml and 1 ml aliquots were added to monolayers that had been pre-washed 4 times with tissue culture medium without antibiotics, with each strain being tested in triplicate. To confirm initial cell numbers, 20 µl of the bacterial suspension was diluted with 180 µl PBS and analysed by FCM. After incubation for 2 h at 37°C in 5% CO_2_, non-adhered bacteria were removed by aspiration and monolayers were gently washed 3 times each with 1 ml tissue culture medium without antibiotics, then dislodged with 1 ml trypsin/EDTA. After serial dilution in PBS, the number of adhered bacteria was analysed by FCM and expressed as a percentage of the initial added bacteria for each strain.

FCM experiments were performed on a Cytomics FC500 MPL (Beckman Coulter). Bacterial viability of each strain was assessed using propidium iodide (PI, 1 mg/ml, Invitrogen Molecular Probes). After adhesion experiments, the bacteria-HT29 cell complexes were quantified and morphological scatter information was obtained by measuring 488 nm forward scatter (FSC) and side scatter (SSC) signals. FCM data were analysed using Flowjo version 7.6.5 (Treestar).

## Results

### The *L. johnsonii* smooth variant shows an altered phenotype

**Figure 1:**
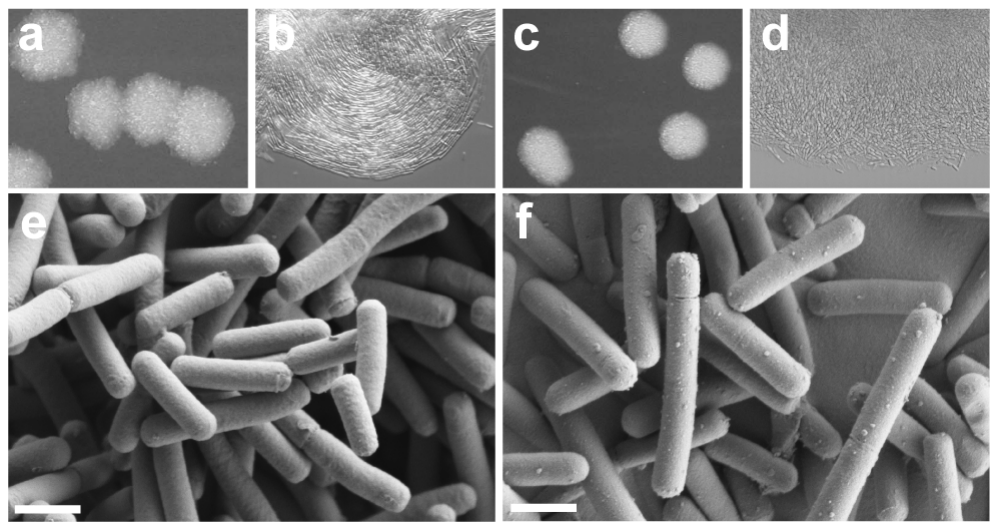
Morphology of *L. johnsonii* wild type and spontaneous mutant strains. Wild type rough (FI9785, a, b, e) and smooth (FI10386, c, d, f) colony variants showing colony phenotype on agar (a, c), organisation within colonies (b, d, magnification ×40) and with SEM (e, f, bar = 1 µm).

The *L. johnsonii* smooth variant FI10386 presented several morphological changes compared to the wild type strain FI9785 (Figure 1). On agar medium, colonies were whiter, smooth-edged and slightly domed compared to the flat, irregularly edged, low opacity colonies of the wild type strain. At greater magnification, the wild type colonies showed a higher degree of organisation with similar sized and shaped cells aligning in rows along the longitudinal axis, while cells of the smooth colonies had a looser distribution and a number of misshapen cells. Viewed by SEM, the two strains seemed similar but the smooth variant showed a less dimpled surface and a high frequency of small lumps on the surface. In addition, cells of FI10386 appeared slightly larger than those of FI9785, and this observation was supported by the FCM data. There was no difference in growth rate in liquid culture between the two strains but the smooth mutant maintained a more even distribution throughout the suspension, while in the wild type there was a tendency for a proportion of the cells to settle. When cells were centrifuged and resuspended, the wild type strain pellet was slightly glutinous and did not resuspend in buffer as freely as the smooth variant. Other colonies showing a smooth phenotype were observed in plate culture of the wild type strain at a frequency of approximately 0.5% of the colony population.

### 2-D gel proteomic analysis revealed an altered protein in the smooth colony variant

**Figure 2:**
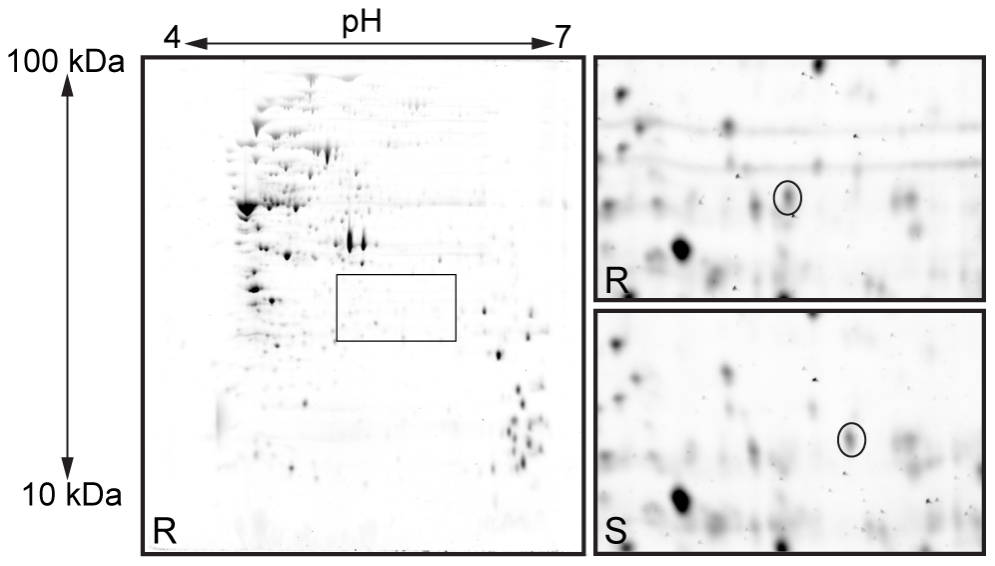
Identification of a protein change associated with the smooth colony phenotype. 2-D gel electrophoresis of proteins extracted from wild type rough (FI9785, R) and smooth colony variant (FI10386, S) strains of *L. johnsonii*. Left, whole gel image of proteins extracted from wild type showing location of enlarged section in top right; bottom right, equivalent section from gel of smooth variant. The spots corresponding to EpsC are circled.

**Figure 3:**
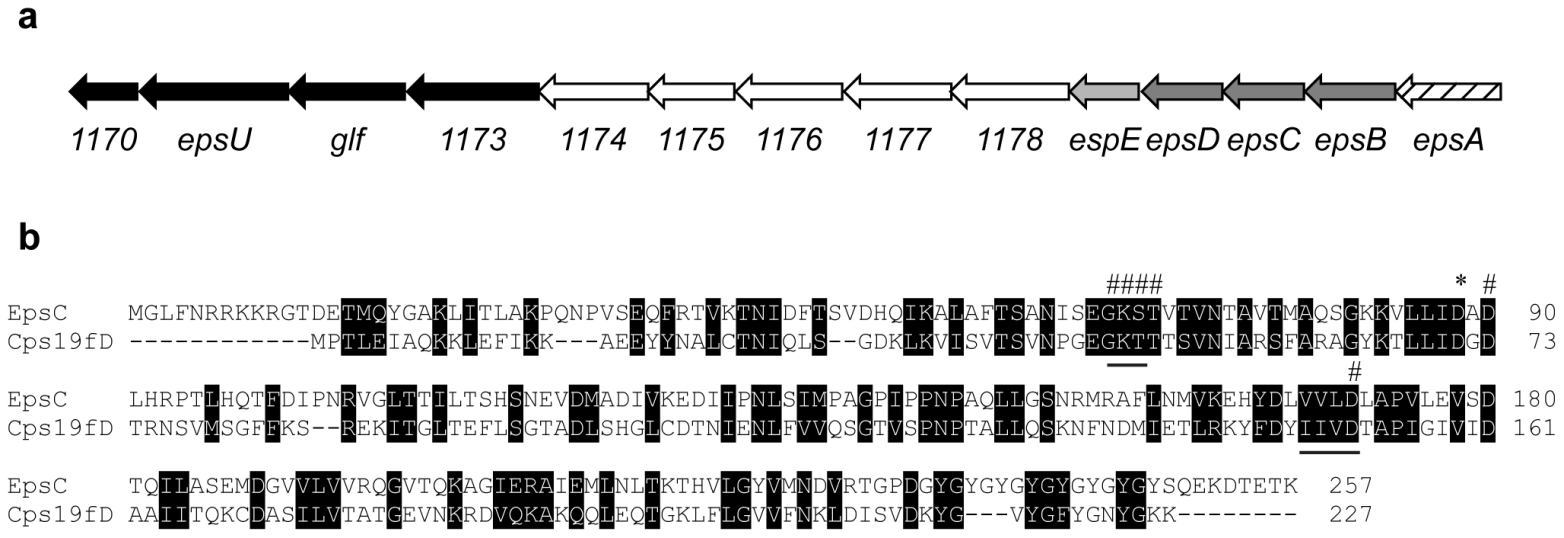
Location of *epsC* in a predicted exopolysaccharide biosynthesis cluster and features of its amino acid sequence. a) Putative *eps* operon of *L. johnsonii* FI9785 showing predicted gene functions. Striped, transcriptional regulation; dark grey, chain length determination, polymerisation and regulation; light grey, priming glycosyltransferase; white, glycosyltransferases; black, polymerisation and export. b) Alignment of the EpsC protein with CpsD from *Streptococcus pneumoniae* (Q54520); #, amino acids involved in the nucleotide binding site cd02035, *, D88 mutated to N88 in smooth variant FI10386, regions with similarity to the Walker A motif (GKT/S) and the Walker B motif (hhhD) are underlined.

Proteomic analysis of soluble protein extracts from the rough wild type and the smooth variant identified a protein spot which was present in one position in FI9785 but had moved to a more basic position in FI10386 (Figure 2). Using peptide mass fingerprinting (Table 2) both of these spots were identified as a putative tyrosine-protein kinase, the gene of which resides in a predicted *eps* cluster (Figure 3a). The first 4 ORFs of the proposed *eps* cluster identified in *L. johnsonii* FI9785 were amplified from the smooth variant and sequenced. This revealed a single substitution (G to A) at position 262 of the *epsC* coding strand, changing an aspartic acid residue D88 to asparagine N88 (Figure 3b) in the gene product. The theoretical pI/MW of this mutant EpsC^D88N^ (pH 6.60/28274.53 Da) agreed with the proteomic data, showing a shift in pI and molecular weight from the theoretical values of the wild type EpsC (pH 6.31/28275.52 Da). Also the corresponding masses for the resulting tryptic peptides were found to be present in the MALDI-TOF data (Table 2); 2584.39 Da for sequence VLLI**D**ADLHRPTLHQTFDIPNR in the wild type and 2583.43 Da for predicted sequence VLLI**N**ADLHRPTLHQTFDIPNR in the mutant. EpsC has a region with similarity to several domains involved in EPS synthesis (e.g. eps-fam [TIGR01007] capsular exopolysaccharide family 1.85 e-55, pepcterm_TyrKin [TIGR03018] exopolysaccharide/PEP-CTERM locus tyrosine autokinase 2.85 e-42, EpsG [TIGR03029] chain length determinant protein tyrosine kinase EpsG 1.14 e-35 and eps_trans_fam [TIGR01005] exopolysaccharide transport protein family 2.48 e-26) as well as similarity to the superfamily P-loop containing nucleoside triphosphate hydrolases, including putative nucleotide phosphate binding motifs with similarity to the Walker A motif (GxxxxGKS/T) and the Walker B motif (hhhhD/E where h is a hydrophobic residue). The same area also has similarity to the ArsA ATPase (cd02035). There are a string of GY motifs at the C-terminus, (GY)_8_, which may be the target for tyrosine phosphorylation. When compared to the experimentally-confirmed protein-tyrosine kinases CpsD2 (AAD10173, [31]) and Cps19fD (Q54520, [32], Figure 3b) from *Streptococcus pneumoniae*, EpsC shows amino acid homologies of 40.5% consensus/25.3% identity and 42.4% consensus/26.1% identity respectively. This similarity includes good conservation of residues identified as highly conserved among similar proteins at the Walker A and B motifs and the tyrosine-rich region ([32], Figure 3b). The identified mutation D88N is part of a region (L/I/V, L/I/V, I/V/L, D, X, D; where X is commonly an A) 21 amino acids downstream from the Walker A motif (Figure 3b). Alignments showed this region to be highly conserved among the homologous proteins from lactobacilli (data not shown). An alignment with the ArsA ATPase nucleotide binding site shows a similar motif VLLVSTD where the final D residue, D45 (corresponding to D90 in EpsC), is involved in the coordination of Mg^2+^ at the nucleotide binding site and is thought to be essential for ATPase activity [33].

**Table 2:** Masses obtained from MALDI-TOF analysis for identification of putative tyrosine protein kinase EpsC.

L. johnsonii FI9785		L. johnsonii FI10386	
m/z	Sequence relating to mass	m/z	Sequence relating to mass
751.39		701.27	
**762.43**	GLFNRR	**762.43**	GLFNRR
795.37		796.50	
798.51		798.51	
823.37		827.49	
826.52		**838.44**	AFLNMVK
827.50		842.51	
833.31		843.28	
**838.44**	AFLNMVK	843.52	
842.51		844.03	
844.02		904.43	
904.43		987.57	
987.57		1014.54	
1001.58		1045.56	
1014.53		1192.83	
1045.56		1201.60	
1201.60		1206.85	
**1216.52**	GTDETMQYGAK	**1308.68**	ALAFTSANISEGK
1291.60		**1419.71**	THVLGYVMNDVR
1296.52		**1517.77**	TNIDFTSVDHQIK
1297.55		**1738.91**	STVTVNTAVTMAQSGKK
**1308.67**	ALAFTSANISEGK	**1841.05**	LITLAKPQNPVSEQFR
**1419.71**	THVLGYVMNDVR	2211.11	
**1517.76**	TNIDFTSVDHQIK	2283.18	
**1841.03**	LITLAKPQNPVSEQFR	**2583.43**	VLLI**N**ADLHRPTLHQTFDIPNR *
2211.10		2691.30	
2230.22		2788.43	
2283.18		3338.70	
**2584.39**	VLLI**D**ADLHRPTLHQTFDIPNR		
2806.28			
Mascot score 89		Mascot score 76	

Mascot Scores greater than 52 are significant. Enboldened masses were used to identify the tyrosine protein kinase;

* , predicted sequence.

**Figure 4:**
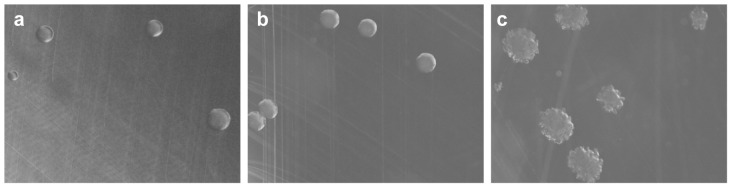
Effect of the expression of the wild type *epsC* gene in the smooth colony strain. Morphology of smooth colony variants containing the *L. johnsonii* expression construct with no insert (a) or containing the mutant *epsC^D88N^* (b) or the wild type *epsC* (c).

In order to investigate the contribution of the mutated *epsC^D88N^* sequence to the smooth colony phenotype, a novel expression vector was constructed based on the native small plasmid p9785S and used to constitutively express both the wild type *epsC* sequence and the mutant *epsC^D88N^* version at a high level in the smooth variant FI10386. Both the empty vector control and the transformant expressing the mutated *epsC^D88N^* sequence maintained the smooth colony phenotype, while co-expression of the wild type *epsC* gene in the smooth mutant (strain FI10773) caused a reversion to the rough colony phenotype (Figure 4). Like the wild type, cells of FI10773 were slightly problematic to resuspend in buffer after centrifugation.

**Figure 5:**
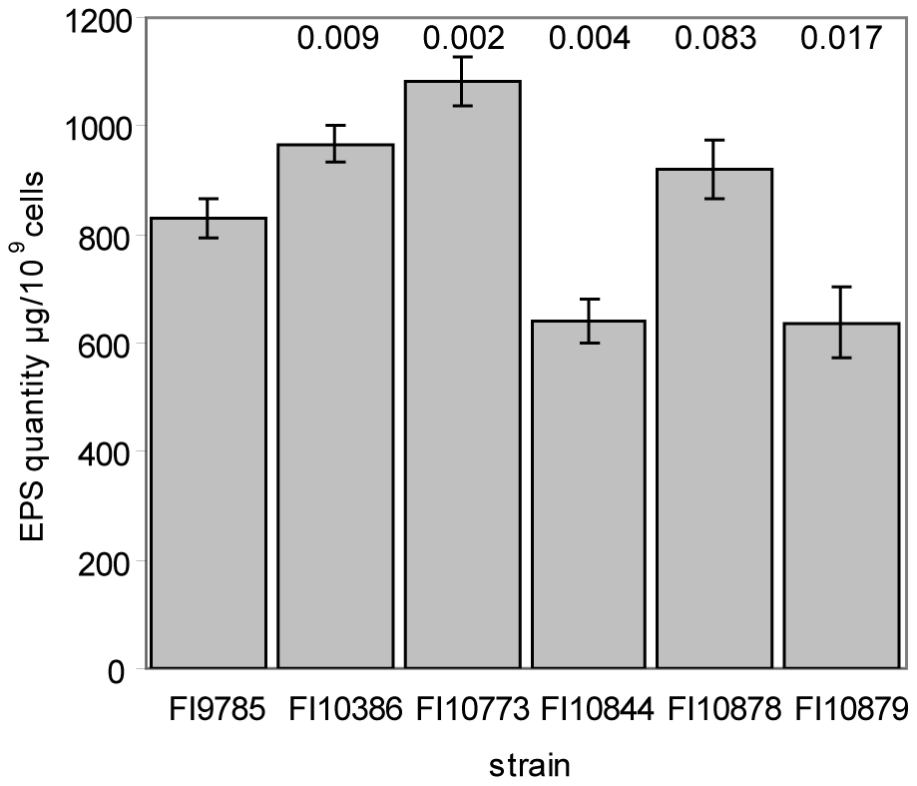
Total sugar content of *L. johnsonii* strains. Results are the mean of triplicate measurements +/− standard deviation, figures above bars represent the p value calculated using an independent t-test with unequal variance, comparing each strain to the wild type.

An analysis of total EPS production by GC demonstrated increased levels of EPS in the smooth variant FI10836 (Figure 5). Co-expression of the wild type *epsC* gene in the smooth variant produced a slight further increase in EPS content, despite its rough colony phenotype. This result indicates that the colony phenotype is not related solely to the amount of EPS present.

### Analysis of the predicted *eps* cluster reveals similarities to genes encoding EPS biosynthesis and control

The predicted *eps* cluster is located on the *L. johnsonii* FI9785 chromosome (NC_013504) and contains 14 ORFs with homology to proteins involved in EPS biosynthesis (Table 3). The first ORF, *epsA*, is predicted to be involved in transcriptional regulation and contains similarity to a LytR domain. Based on BlastP analysis, the gene *epsB* codes for a protein with homology to capsular polysaccharide biosynthesis proteins and polymerisation and chain length determination proteins; similarly, as noted above, EpsC is predicted to be a tyrosine protein kinase and EpsD a protein-tyrosine-phosphate phosphohydrolase. These 4 proteins and the predicted priming glycosyltransferase EpsE, a putative undecaprenyl-phosphate galactosephosphotransferase, are all highly similar to homologous proteins encoded by the *eps* clusters of *L. johnsonii* NCC533 [19] and *L. johnsonii* ATCC33200. They are followed by 5 ORFs predicted to code for glycosyltransferases - none of these show strong similarity to *L. johnsonii* NCC533 or ATCC33200 *eps* operon-encoded glycosyltransferases but they are highly similar to proteins from *L. johnsonii* pf01 [34]. Some similarity to the gene products from the *L. johnsonii* NCC533 *eps* cluster is regained at *FI9785_1173*, a putative oligosaccharide repeat unit polymerase while *FI9785*_*1172* and *FI9785_1171* (predicted *glf* UDP-galactopyranase mutase and *epsU* oligosaccharide translocase respectively) have highly similar matches in both *L. johnsonii* NCC533 and ATCC33200. However, the clusters from the latter two genomes include an ORF between the last two genes which is absent in *L. johnsonii* FI9785. The final ORF *FI9785_1170*, coding for a predicted EPS biosynthesis protein, is followed by a transposase pseudo gene.

**Table 3:** *L. johnsonii eps* gene cluster analysis.

ORF	location (complement)	Protein ID	Predicted product	Top conserved domain (E value)/superfamily	Top BlastP match (E value)
*epsA*	1113642–1114646	YP_003293311	transcriptional regulator	PRK09379 LytR (5.3e-79)/LytR_cpsA-psr	HP*, *L. johnsonii* NCC533 (e0)
*epsB*	1112772–1113635	YP_003293310	polymerisation and chain length determination protein	TIGR01006 Polys_exp_MPA1 (4.3e-27)/Wzz	Capsular polysaccharide biosynthesis protein, *L. johnsonii* DPC6026 (e0)
*epsC*	1,111,988–1,112,761	YP_003293309	tyrosine protein kinase	TIGR01007 eps_fam (1.9e-55)/P-loop NTPase	Tyrosine-protein kinase, *L. gasseri* (1e-164)
*epsD*	1,111,211–1,111,981	YP_003293308	protein-tyrosine-phosphate phosphohydrolase	COG4464 CapC (2.9e-68)/-	Manganese-dependent protein-tyrosine-phosphatase *L. johnsonii* pf01 (e0)
*epsE*	1,110,522–1,111,181	YP_003293307	undecaprenyl-phosphate galactosephospho-transferase	Pfam02397 Bac_transf (3.1e-93)/Bac_transf	Phosphor-glycosyltransferase *L. johnsonii* ATCC11506 (1e-152)
*1178*	1,109,378–1,110,511	YP_003293306	glycosyltransferase	cd03808 GT1_cap1E_like (1.4e-87)/Glycosyltransferase GTB_type	Glycosyltransferase *L. johnsonii* pf01 (e0)
*1177*	1,108,360–1,109,388	YP_003293305	glycosyltransferase	cd00761 Glyco_tranf_GTA_type (1.7e-28)/Glyco_tranf_GTA_type	Beta-1,3-glucosyltransferase *L. johnsonii* pf01 (e0)
*1176*	1,107,323–1,108,342	YP_003293304	glycosyltransferase	Pfam00535 Glycos_transf_2 (7.3e-39)/Glyco_tranf_GTA_type	Glycosyltransferase *L. johnsonii* pf01 (e0)
*1175*	1,106,483–1,107,310	YP_003293303	glycosyltransferase	Pfam04488 Gly_transf_sug (1.9e-16)/Gly_transf_sug	Polysaccharide biosynthesis protein CpsM *L. johnsonii* pf01 (e0)
*1174*	1,105,437–1,106,486	YP_003293302	glycosyltransferase	PRK09814 beta-1,6-galactofuranosyltransferase (7.8e-120)/Glycosyltransferase GTB_type	Putative Galf transferase *L. johnsonii* pf01 (1e-141)
*1173*	1,104,178–1,105,437	YP_003293301	oligosaccharide repeat unit polymerase	-/-	Putative Galf transferase *L. johnsonii* pf01 (e0)
*glf*	1,103,044–1,104,156	YP_003293300	UDP-galactopyranase mutase	COG0562 Glf (4.6e-166)/GLF	UDP-galactopyranase mutase, *L. johnsonii* NCC533
*epsU*	1,101,617–1,103,038	YP_003293299	oligosaccharide translocase	Pfam01943 Polysacch_synt (1.2e-34)/MatE	Membrane protein *L. johnsonii* pf01 (e0)
*1170*	1,100,951–1,101,595	YP_003293298	exopolysaccharide biosynthesis protein	-/DUF1919	Exopolysaccharide biosynthesis protein *L. johnsonii* pf01 (2e-138)

* , hypothetical protein.

The *epsE* gene of *L. johnsonii* FI9785 has similarity to several conserved domains associated with priming glycosyltransferase activity (Undecaprenyl-phosphate galactose phosphotransferase domains WbaP_sugtrans [TIGR03022] 1.93 e-77, Wca_J [TIGR03023] 7.13 e-63, PRK15204 7.7 e-56). When compared to priming glycosyltransferases whose activity has been demonstrated experimentally, there are good levels of similarity across the length of the protein, with percentage similarities of 51.5% consensus/40.5% identity to priming glycosyltransferase EspD from *Lactococcus lactis* NIZO B40 (AAC45231 [35]), 74.5% consensus/63.2% identity to EpsE from *Lactobacillus delbrueckii* subsp *bulgaricus* (AAG44709 [17]) and 54.2% consensus/39.2% identity to EpsE from *Streptococcus thermophilus* Sfi6 (AAC44012 [36]).

### Deletion of the *epsE* gene affects aggregation and reduces EPS content

**Figure 6:**
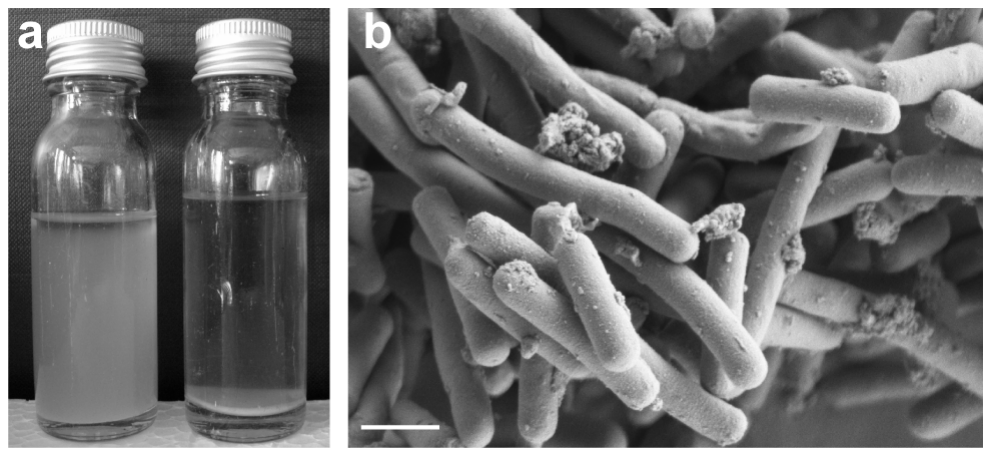
Effect of the deletion of *epsE* on *L. johnsonii* morphology. a) Aggregation of *L. johnsonii* Δ*epsE* FI10844 (right) compared to *L. johnsonii* FI9785 (left) after 24 h growth at 37°C. b) SEM of FI10844 cells, bar = 1 µm.

To investigate the role of the putative priming glycosyltransferase gene *epsE* on the phenotype of *L. johnsonii*, a derivative of strain FI9785 was produced where *epsE* had been deleted. This strain was then complemented with the wild type gene in the sense orientation, while a construct with the gene in the antisense orientation provided a negative control. All of these strains retained the rough colony phenotype; however the deletion of *epsE* resulted in a strong aggregation phenotype demonstrated by the rapid sedimentation of the cells (Figure 6a). This effect was complemented by the plasmid carrying the sense gene but not by the equivalent plasmid carrying the gene in the antisense orientation (data not shown). There were no striking differences compared to the wild type when viewed by SEM, although the *ΔepsE* knockout strain tended to have a higher amount of the extracellular material that was also seen in SEM of strains FI9785 and FI10386 (Figure 6b). No pili were observed on cells of the wild type, smooth colony variant or *ΔepsE* strains (data not shown).

Total sugar analysis showed clear differences in EPS content after manipulation of the *epsE* gene (Figure 5). Overall, the Δ*epsE* mutant FI10844 and strain FI10879 expressing the antisense *epsE* control vector produced less EPS than the wild type, while EPS synthesis in the *ΔepsE* complemented strain FI10878 was restored to levels slightly above those of the wild type. Although this indicates an important role for the *epsE* gene in EPS biosynthesis, its deletion did not completely abolish EPS production.

### Changes in EPS production affect adhesion to tissue culture cells

**Figure 7:**
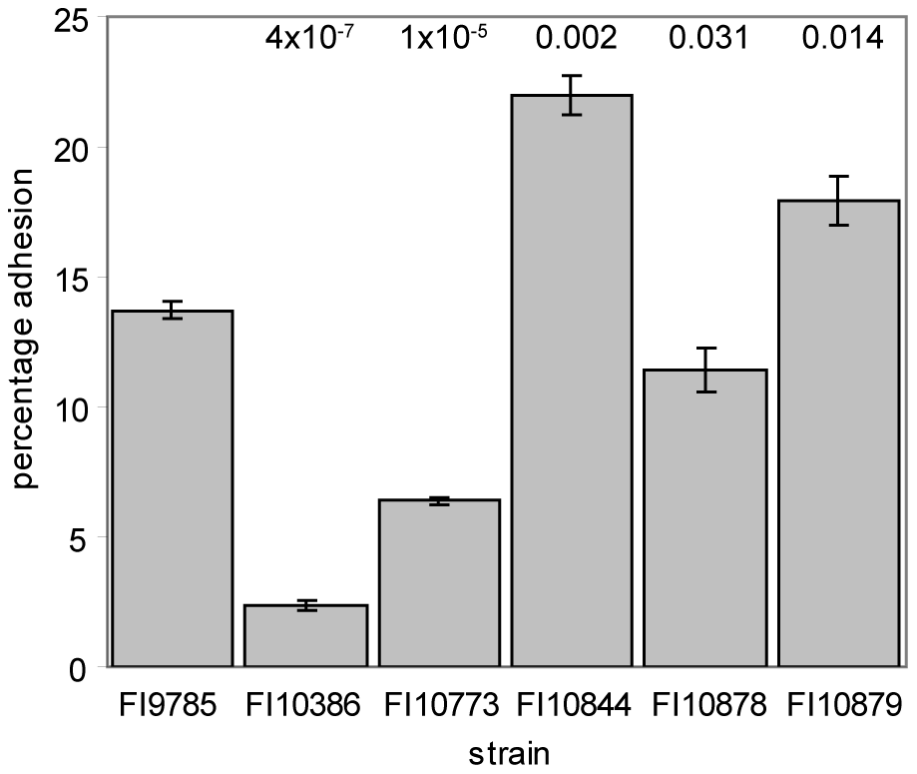
Adhesion of *L. johnsonii* strains to HT29 monolayers. Results are the mean of triplicate experiments with three replicates per experiment +/− standard deviation, figures above bars represent the p value calculated using an independent t-test with unequal variance, comparing each strain to the wild type.

To investigate the impact of alterations in the *eps* genes on adhesion, the ability of the mutant and engineered strains to adhere to HT29 cells was assessed. FCM was used to accurately enumerate bacteria in suspension and bacteria adhering to HT29 cells. The percentage adhesion of the wild type strain FI9785 was 13.7+/−0.2% of the total bacteria added. Adhesion of the smooth colony variant FI10386 was notably reduced to less than 20% of the wild type level (2.4+/−0.2%, Figure 7). Co-expression of the wild type *epsC* gene (strain FI10773) gave a slight increase in adhesion, but levels were still less than half of the wild type values. In contrast, the Δ*epsE* autoaggregating strain FI10844 showed a clear increase in adhesion to approximately 160% of the wild type level (22.0+/−0.8%). The wild type phenotype could largely be restored by complementation with a plasmid expressing the *epsE* gene in the sense orientation while the negative control containing *epsE* in the antisense orientation maintained higher levels of adhesion than the wild type, although not as high as the deletion mutant FI10844.

## Discussion

Analysis of a spontaneous variant of *L. johnsonii* revealed a link between a smooth colony phenotype and EPS biosynthesis. The *eps* cluster identified during genome annotation [1] shows similarity to clusters for heteropolysaccharide production in a range of lactic acid bacteria [8], [13], [14], [16], [17], [18], including genes for regulation, chain length determination, biosynthesis, polymerisation and export. The product of the *epsC* gene is predicted to be a tyrosine protein kinase; these have been associated with the production of EPS required for virulence in selected bacterial pathogens [37]. Other tyrosine protein kinases from polysaccharide biosynthesis clusters have been shown to be involved in a phosphorylation complex associated with the regulation of EPS synthesis, with proteins with similarity to EpsB, EpsC and EpsD cooperating to control polymerisation and export [8], [38], [39]. Analysis of the autophosphorylating protein-tyrosine kinase in the capsular polysaccharide biosynthesis cluster of *Streptococcus pneumoniae* (CpsD) demonstrated that deletion of the gene or a mutation in the Walker A motif disabled encapsulation [32]. The authors proposed that an interaction between CpsC and CpsD requiring the ATP binding motif enabled capsular polysaccharide biosynthesis/polymerisation, tyrosine phosphorylation of CpsD interfered with the interaction leading to negative regulation, and dephosphorylation of CpsD by CpsB allowed the interaction to resume and polysaccharide biosynthesis/polymerisation to continue. However they also showed that removal of the tyrosine repeat domain of CpsD gave a similar result to removal of the entire gene, with a reduction in capsular polysaccharides, indicating that this domain might be important in the complex interaction in addition to being a phosphorylation target [40]. In another strain of *S. pneumoniae*, researchers concluded that CpsB, CpsC, CpsD and ATP form a stable complex that regulates capsule production [31]. In *Streptococcus thermophilus* the tyrosine kinase (EpsD) was also required for EPS synthesis, and activity of the priming phosphogalactosyltransferase EpsE was not present in deletion derivative strains of either *epsC* or *epsD*, indicating that this may be the target for the phosphorylation complex in this species [41]. The spontaneous mutation in the *L. johnsonii* FI9785 protein tyrosine kinase *epsC* gene affects an amino acid in a conserved area downstream of the Walker A motif. A similar sequence is also conserved in tyrosine protein kinases of Gram-negative bacteria [37], while analysis of the ArsA ATPase has identified an aspartate residue in this area (D45) as being important for ATPase activity [33]. This residue in particular is highly conserved in a range of bacteria, and as D88N in the FI10386 smooth colony mutant is located only 2 amino acids away from this residue it is possible that the mutation has an effect on its function. The smooth colony phenoype suggested that the *epsC^D88N^* mutation increased the production of EPS, and total sugar analysis confirmed that EPS content was higher in FI10386. This suggests that the mutation may stabilise the interaction between EpsC and EpsD and/or ATP in some way to give increased production.

Co-expression of the wild type *epsC* gene and the mutant *epsC^D88N^* also produced high levels of EPS, but combined with the wild type rough phenotype. This indicates that the quality of EPS may play an important role in the rough phenotype. Examination of the capsular biosynthesis genes in Type Ia Group B *Streptococcus* found that CpsIaC and CpsIaD were involved in the control of both chain length and the number of polysaccharide molecules of EPS [38]. Mucoid strains of *S. pneumoniae* produced by CpsD tyrosine-deficient mutants also showed a reduction in the molecular weight of the capsular polysaccharide [40]. It is possible that variation in chain length may be responsible for the observed phenotypic changes in the *epsC^D88N^* mutant and further investigation of this phenomenon may give insight into the mechanism of EPS polymerisation and regulation in *L. johnsonii*.

EpsE is predicted to act as the priming glycosyltransferase which adds the first sugar to the phosphorylated lipid carrier [14], [41]. Deletion of the genes encoding the priming glycosyltransferases of *Streptococcus thermophilus*, *Lactococcus lactis* or Type Ia Group B *Streptococcus* abolished heteropolysaccharide production [35], [38], [41]. Deletion of the *epsE* gene from *L. johnsonii* FI9785 led to a large increase in autoaggregation, a decrease in EPS production and an increase in the adhesion of the bacteria to HT29 cells. However, EPS was still present, suggesting that there may be more than one form of EPS produced by *L. johnsonii* FI9785. This is not unprecedented; the mutation of the *welE* gene of *Lactobacillus rhamnosus* GG encoding a predicted undecaprenyl-phosphate galactose phosphotransferase prevented the biosynthesis of a galactose-rich EPS without affecting a second glucose-rich EPS, and resulted in a concomitant increase in biofilm formation and adhesion to mucus and Caco-2 cells [14]. Two different EPS polymers were also found in *Lactobacillus plantarum* EP96 [28]. Preliminary results confirm the presence of a second EPS (Dertli et al, in preparation) and a more detailed examination of the EPS in *L. johnsonii* FI9785 and its derivatives should provide further insights into sugar content and chain length.

Mutations in the *eps* gene cluster affected both aggregation and adhesion. Aggregation may affect both colonisation and interactions with other bacteria [4], [11]. For example, a *Lactobacillus crispatus* strain with an aggregating phenotype had improved adhesion and gastrointestinal persistence compared to its non-aggregating mutant [42]. The strain was also often recovered from *in vivo* studies in association with other bacteria, indicating that aggregation affects its relationship with other microbes. However, two *L. johnsonii* strains isolated from fermented milk products showed a higher adhesion to HT29 cells than the NCC533 strain but a lower level of autoaggregation [43], indicating that a high aggregation phenotype cannot be taken as a reliable indicator of high adhesion *in vitro*. The relationship of the new aggregating phenotype of the *epsE* deletion mutant with changes in EPS production might go some way to explain this discrepancy, as aggregation or adhesion can be caused by a multitude of factors (EPS, hydrophobicity, protein factors and lipoteichoic acids) that may positively or negatively affect adherence to other cells [4], [7]. Deletion of the EPS biosynthesis cluster in *L. johnsonii* NCC533 produced a slight increase in residence time in the murine gut [9], which may reflect changes in the adhesion to or interaction with the cells or mucus of the gastrointestinal tract. Both elongation factor Tu and lipoteichoic acid have been shown to be involved in the binding of *L. johnsonii* NCC533 to mucin and/or human cells [44], [45]. It is likely that changes in the thickness or nature of the EPS layer might make such adhesive factors more exposed and improve the accessibility of such adhering molecules to intestinal cells, as has been suggested previously [14], in addition to affecting charge or hydrophobicity/hydrophilicity characteristics. Insertional inactivation of the priming glycosyltransferase of *Lactobacillus rhamnosus* GG revealed the presence of fimbriae which could potentially affect adhesion [14], but these were not observed in *L. johnsonii* FI9785 or its *ΔepsE* derivative. However, the genome of *L. johnsonii* FI9785 does contain 2 genes coding for proteins with strong similarity to aggregation promotion factor proteins located on the cell surface of other *L. johnsonii* strains [46]. Although a reduction in EPS may allow for better adhesion facilitated by surface molecules, conversely, its presence might aid colonisation, as EPS has also been shown to be important in enabling *Bifidobacterium breve* to prevent the induction of the host immune response [10]. Deletions of genes involved in the synthesis of homopolysaccharide and oligosaccharide EPS in *Lactobacillus reuteri* had negative effects on aggregation, biofilm formation, competition and colonisation [11], indicating that in some cases EPS is a positive mediator of interactions rather than an obscuring coating. The nature of EPS and its interactions with other surface factors may also play a role, as suggested by the increased adhesion of the strain expressing both *epsC^D88N^* and *epsC*. The extent of bacterial aggregation may also impact on how the bacteria interact with the host tissue. It is important to note that the methodology to assess aggregation and adhesion in different systems can play a key factor in the results. In addition, adhesion of *L. johnsonii in vitro* does not always mimic survival and colonisation in the host: *L. johnsonii* NCC533 and *Lactobacillus paracasei* showed similar levels of adhesion to Caco-2 cells *in vitro* but only *L. johnsonii* was able to colonise the intestine of germ free mice [47]. It will be essential to assess the effect of these mutations in poultry *in vivo* and in competition with other microflora before any impact of the EPS on the competitive exclusion capacity of *L. johnsonii* can be inferred.

We have not determined whether all smooth colony phenotypes seen in *L. johnsonii* FI9785 culture are caused by changes in the *eps* cluster genes. However, the frequency of appearance of the smooth colony phenotype might suggest a deliberate ploy to maintain variability in the population. A spontaneous variant of *Lactobacillus rhamnosus* ATCC9595 with altered EPS production has also been described [18], and other instances of instability of EPS production have been noted [13]. It could be that the ability to produce occasional mutants with variations in EPS quantity and/or quality may render the population more able to survive varying environments and challenges without affecting the intrinsic metabolism of the cell.

This study has demonstrated that alterations in the proteins encoded by a putative *eps* gene cluster affect colony phenotype, EPS production and surface characteristics of *L. johnsonii*. These strains represent an opportunity for the greater understanding of EPS biosynthesis and the impact of EPS on colonisation and pathogen exclusion.
